# Technological, Ecological, and Energy-Economic Aspects of Using Solidified Carbon Dioxide for Aerobic Granular Sludge Pre-Treatment Prior to Anaerobic Digestion

**DOI:** 10.3390/ijerph20054234

**Published:** 2023-02-27

**Authors:** Joanna Kazimierowicz, Marcin Dębowski, Marcin Zieliński

**Affiliations:** 1 Department of Water Supply and Sewage Systems, Faculty of Civil Engineering and Environmental Sciences, Bialystok University of Technology, 15-351 Bialystok, Poland; 2 Department of Environment Engineering, Faculty of Geoengineering, University of Warmia and Mazury in Olsztyn, 10-720 Olsztyn, Poland

**Keywords:** aerobic granular sludge (AGS), anaerobic digestion (AD), pre-treatment, solidified carbon dioxide (SCO_2_), methane fermentation, biogas, methane, process optimization

## Abstract

The technology of aerobic granular sludge (AGS) seems prospective in wastewater bio-treatment. The characteristics as well as compactness and structure of AGS have been proved to significantly affect the effectiveness of thus far deployed methods for sewage sludge processing, including anaerobic digestion (AD). Therefore, it is deemed necessary to extend knowledge on the possibilities of efficient AGS management and to seek viable technological solutions for methane fermentation of sludge of this type, including by means of using the pre-treatment step. Little is known about the pre-treatment method with solidified carbon dioxide (SCO_2_), which can be recovered in processes of biogas upgrading and enrichment, leading to biomethane production. This study aimed to determine the impact of AGS pre-treatment with SCO_2_ on the efficiency of its AD. An energy balance and a simplified economic analysis of the process were also carried out. It was found that an increasing dose of SCO_2_ applied in the pre-treatment increased the concentrations of COD, N-NH_4_^+^, and P-PO_4_^3−^ in the supernatant in the range of the SCO_2_/AGS volume ratios from 0.0 to 0.3. No statistically significant differences were noted above the latter value. The highest unit yields of biogas and methane production, reaching 476 ± 20 cm^3^/gVS and 341 ± 13 cm^3^/gVS, respectively, were obtained in the variant with the SCO_2_/AGS ratio of 0.3. This experimental variant also produced the highest positive net energy gain, reaching 1047.85 ± 20 kWh/ton total solids (TS). The use of the higher than 0.3 SCO_2_ doses was proved to significantly reduce the pH of AGS (below 6.5), thereby directly diminishing the percentage of methanogenic bacteria in the anaerobic bacterial community, which in turn contributed to a reduced CH_4_ fraction in the biogas.

## 1. Introduction

The methods for sewage sludge processing are well-known and widely implemented on a technical scale. They have been optimized for the most commonly applied wastewater treatment solutions based on the use of suspended conventional activated sludge (CAS) [1]. A novel, alternative, and competitive technology is offered by the use of granular activated sludge (AGS) [2]. The strengths and advantages of this solution have spurred the interest of researchers, operators, and designers, which has consequently led to a growing number of commercial-scale installations [3,4]. The use of AGS has been shown to allow simplifying the technology, increasing the resistance of the biological system to the variability of wastewater composition, shortening the reactor operation cycle, resigning from the need to ensure variable oxygen conditions in order to remove nutrients, and simplifying the methods of excess sludge separation [5]. The AGS-based wastewater treatment systems are now widely accepted as promising and future-proof solutions due to their high level of technical readiness, optimized processes for growing stable granules, and established and verified parameters of pollutant biodegradation [6]. 

Little is, however, known about the conversion and neutralization of excess AGS. Given the various properties and characteristics of AGS, the unitary processes applied today need to be verified and adjusted to the substrate featuring different chemical compositions and properties [7]. Aerobic granules are a type of aggregated, compact microbiological structure formed upon the self-immobilization of bacteria [8]. Unlike floccules, they have clearly outlined quasi-spherical shapes, larger dimensions, and a more compact structure [9]. These features of the biological structure significantly diminish the efficiency of the known methods for sewage sludge processing. A need arises, then, to seek effective solutions for the technologically and economically viable management of excess AGS. One of the most justified and environment-friendly methods for sewage sludge neutralization is to subject it to methane fermentation (MF) [10], which ensures reduced susceptibility to putrefaction, partial hygienization, volume reduction, and also enables the recovery of biogas with a high methane content [11]. These effects may be boosted by sewage sludge pre-treatment, which is especially advisable during methane fermentation of AGS due to the compactness and complex structure of its granules [12].

Dynamically developing pre-treatment technologies aim to induce disruption of the compact structure of sludge and disintegration of microbial cells [13]. These phenomena lead to the release of organic substances and extracellular polymers to the dissolved phase, thereby increasing their availability to anaerobic bacteria [14]. The most frequently deployed disintegration methods include mechanical destruction methods, microwave and conventional heating, ultrasound disintegration, hydrodynamic cavitation, pulsed electric field, high-pressure depolymerization, enzymatic and biological methods, chemical pre-treatment with acids, bases or strong antioxidants, as well as freezing/thawing. Additionally, hybrid methods are in use, namely coupled techniques of disintegration [15]. In most cases, they yield satisfactory technological outcomes associated with anaerobic digestion (AD) effectiveness, including increased biogas yield and methane content of the biogas produced as well as a higher mineralization ratio and organic matter degradation [16]. Most often, however, the energy balance owed to sewage sludge pre-disintegration is negative, meaning that the net energy gain does not compensate for the energy output incurred for the exploitation of pre-treatment facilities [17]. Investment costs and outlays for the service, maintenance, and repairs of disintegration systems are also high [18]. Hence, it is necessary to search for novel, universal, and environment-friendly technologies for sewage sludge pre-treatment that would offer a competitive alternative to the existing methods in terms of both the final outcomes of AD and the economic viability of the entire process.

The use of solidified carbon dioxide (SCO_2_) may prove a prospective technology in sewage sludge processing. It is a natural by-product of natural gas purification processes or can be produced in technologies dedicated to biogas upgrading [19]. Its sources and its production methods correspond to the material recycling idea and are directly in line with the assumptions of the circular economy [20]. They also support the idea of reducing carbon dioxide emissions through its sequestration and use in a closed cycle. The production and use of SCO_2_ in the processes of sewage sludge disintegration entail activities leading to the capture, separation, transport, and long-term retention of CO_2_ in an appropriate, safe place [21]. 

Fragmentation of the granules and subsequent AGS disintegration under SCO_2_ exposure are caused by an increase in the volume of freezing water in the cytoplasm, mechanical damage to the cell wall and membrane, osmotic shock, and destruction of cell organelles. Mechanical damage is also a consequence of the formation of ice crystals in the environment surrounding the cells and inside them, as well as partial loss of the hydration water of proteins, which modifies their properties. The extracellular crystals that expand during the freezing process destroy the microbial cells that lie between them. They also cause damage to biomembranes and modify their properties, which leads to the leakage of intracellular substances into the environment [22]. 

There are no reports in the world literature on the use of SCO_2_ in the AGS pre-treatment process prior to AD. Given the characteristics of AGS and the results obtained during CAS disintegration, as well as taking into account the possibility of SCO_2_ recovery, it seems justified to verify the possibility of implementing this method for AGS pre-treatment. The main goal of this study was to determine the effect of AGS pre-treatment with SCO_2_ on the efficiency of its AD. The impact of this disintegration method on the structure and properties of sewage sludge, changes in the concentration of organic and biogenic compounds in the dissolved phase, taxonomic structure of anaerobic bacteria, and the yield and kinetics of biogas production and methane content were assessed as well. Finally, empirical optimization models were developed, and an energy and economic balance was prepared in order to demonstrate the competitiveness of the pre-treatment technology under study.

## 2. Materials and Methods

### 2.1. Experimental Design

**Figure 1:**
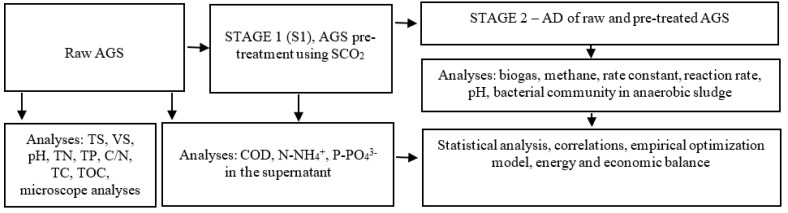
Block diagram of experimental, analytical, and computational works.

The experiment was divided into two stages. In stage 1 (S1), AGS was pre-treated using SCO_2_. In stage 2 (S2), AGS was subjected to AD. S1 and S2 were divided into 6 variants (V), differing in the volume ratio of SCO_2_ to AGS: V1—control, V2—0.1, V3—0.2, V4—0.3, V5—0.4, and V6—0.5. Figure 1 presents the organizational scheme of this research work.

### 2.2. Materials

#### 2.2.1. AGS and Inoculum of the Anaerobic Sludge (AS)

**Figure 2:**
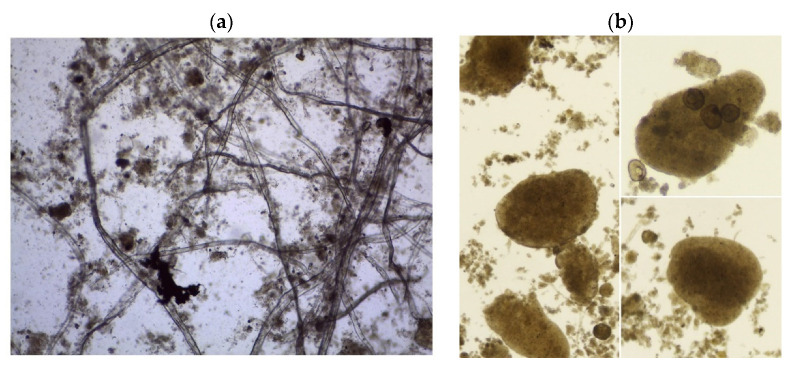
Microscopic image of (**a**) CAS (100× magnification) and (**b**) AGS used in the experiment (100× magnification).

AGS was cultured under laboratory conditions (23 °C, relative humidity 50%). The CAS from the municipal wastewater treatment plant in Rajgród, Poland (53.99434 N, 22.76847 E) with PE = 2500 served as the inoculum. The average capacity of the treatment plant is 400 m^3^/d, and it operates based on the terrace-flow technology with increased nutrient removal. Prior to granule cultivation, the activated sludge was filtered through a sieve with a mesh diameter of 1.0 mm, which allowed the separation of fine suspensions from a mixture of wastewater and activated sludge and enabled achieving a high organic mass of CAS. The AGS culture process was continued for 120 days in a sequencing batch reactor (SBR) [23]. The reinforced PE reactor was equipped with a stirrer, aerating diffuser, oxygen probe, and discharge valve, which enabled decantation at a ratio of 0.33. The rotational speed of the stirrer was 70 rpm. During aeration, the air was supplied to the reactor by a compressor with a capacity of 550 dm^3^/h, maintaining dissolved oxygen concentration at 2.5 mg/dm^3^. In turn, during the filling, mixing, sedimentation, and shutdown phase, the concentration of dissolved oxygen in the reactor was kept below 0.3 mg/dm^3^. The reactor work cycle was 8 h (480 min). The filling and stirring phase lasted 60 min, the aeration phase lasted 270 min, the sedimentation phase lasted 15 min, and the decantation phase lasted 135 min. The reactor was fed with model wastewater composed of: casein peptone (0.452 g/dm^3^), enriched broth (0.304 g/dm^3^), CH_3_COONa (0.300 g/dm^3^), NH_4_Cl (0.242 g/dm^3^), KH_2_PO_4_ (0.032 g/dm^3^), and K_2_HPO_4_ (0.080 g/dm^3^), as well as NaCl (0.014 g/dm^3^), CaCl_2_·6H_2_O (0.015 g/dm^3^), and MgSO_4_·7H_2_O (0.004 g/dm^3^). The mixture of these components ensured the presence of organic carbon, organic nitrogen, ammonium nitrogen, phosphates, and macroelements in the wastewater flowing into the reactor. AGS was produced using the gravimetric selection method as a stress factor. In the first 30 days of the experiment, CAS was adapted to laboratory conditions. Afterward, the exact experiment was performed for the subsequent 90 days, when the gravimetric selection of CAS was successively increased by washing out the slowest sedimenting fractions from the reactor. After 120 days of the experiment, mature granules were obtained, which were used in further research works. Figure 2 shows microscopic images of CAS and AGS. 

The inoculum of fermentation reactors was AS from a closed fermentation chamber (CFC) with a capacity of 7300 m^3^ from a wastewater treatment plant in Białystok, Poland (53.16903 N, 23.08705 E) (Table 1). The plant operated at a temperature of 35 °C, organic load rate (OLR) of 2.0 gVS/dm^3^·d, and hydraulic retention time (HRT) of 21 days. Before being used as an inoculum, the anaerobic sludge was adapted to the experimental conditions of 42 °C for 40 days (HRT = 20 days) and an OLR of 1.0 gVS/dm^3^·d. Table 1 presents the characteristics of CAS, AGS, and AS inoculum. 

#### 2.2.2. SCO_2_

Experiments were conducted with the use of SCO_2_ (Sopel Ltd., Białystok, Poland) in the form of granules 3.0 ± 1.0 mm in diameter. The SCO_2_ used sublimes under pressure below 5.13 atm and temperatures over −56.4 °C. Under atmospheric pressure, its sublimation proceeds at a temperature of −78.5 °C, and sublimation enthalpy is 573 kJ/kg, which makes it ca. 3.3 times more efficient than water ice of the same volume. The SCO_2_ used in the study is a natural, odorless, tasteless, non-toxic, and non-flammable product approved for contact with foodstuffs [24]. 

### 2.3. Experimental Stations

#### 2.3.1. Stage 1

**Figure 3:**
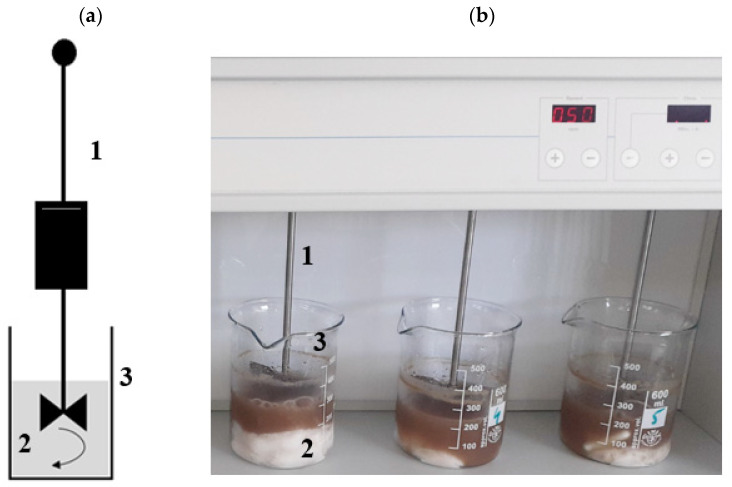
Experimental station in S1: (**a**) scheme; (**b**) photo (1—mechanical stirrer, 2—AGS mixed with SCO_2_, 3—glass reactors).

Experiments were performed using a jar tester (JLT 6, VELP Scientifica, Milano, Italy). AGS having a temperature of 20 °C was poured into glass reactors in single doses of 200 cm^3^, and then an appropriate dose of SCO_2_ was added. The mixture was stirred with a yield of 50 rpm for 20 min. The samples were left for complete SCO_2_ sublimation and then subjected to MF when they had reached a temperature of 20 °C. Figure 3 presents the scheme of an experimental station used in this stage.

#### 2.3.2. Stage 2

**Figure 4:**
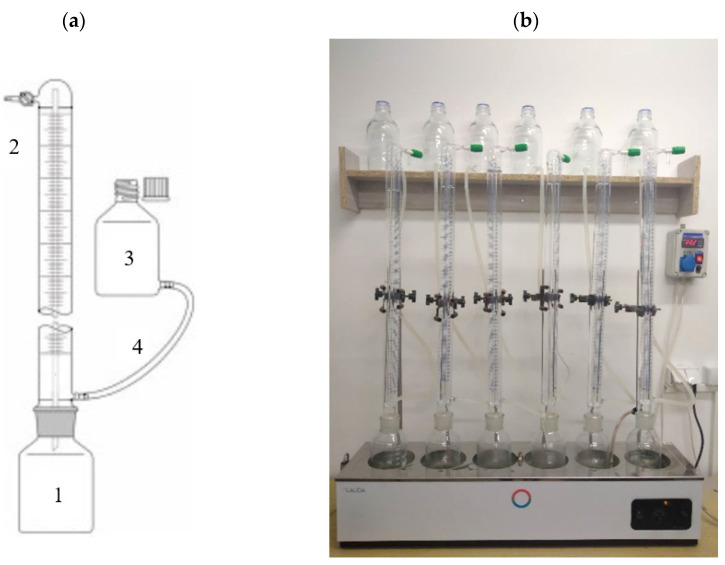
Respirometric measurements: (**a**) scheme of an eudiometer (1—reactor, 2—burette with an internal glass tube for gas transport, 3—pressure equalization tank, 4—connecting tube), (**b**) experimental station photo in S2.

Measurements of the volumes of biogas produced were carried out in a set of eudiometers (Hornik Ltd., Poznań, Poland), the scheme and photo of which are provided in Figure 4. A single eudiometer is made of a reactor with a volume of 1000 cm^3^, connected with a joint to a burette with a volume of 600 cm^3^, inside of which there is a thin capillary. The gas comes out of the fermenter via the capillary and flows to the upper part of the burette, from where it can be taken for analysis through the stub pipe with a valve mounted therein. The principle of measurement is that the gas emitted displaces the liquid from the burette, which flows through the hose to the connected equalization tank to equalize the pressure. A total of 200 cm^3^ of the inoculum were fed to the reactors, followed by the appropriate amount of AGS. In order to remove oxygen from the reaction chambers, the feedstock and the gaseous phase of the respirometer were purged with compressed nitrogen (N40). Nitrogen was introduced via a rubber hose terminated with a stone diffuser placed below the surface of the mixture of inoculum and feedstock for 3 min. The initial OLR was 5.0 gVS/dm^3^ [25]. Respirometers were placed in a temperature control cabinet with a hysteresis of ±0.5 °C. Measurements were conducted at 42 °C. The volume of emitted biogas was read out every day until its production ceased. Measurements of biogas composition were carried out at the end of the process.

### 2.4. Analytical Methods

Contents of TS, VS, and MS were determined with the gravimetric method. Contents of TS in the sludge were determined by drying it to a constant weight at 105 °C, then burning it at 550 °C. The loss after combustion was the VS, according to PN-EN 15935:23022-01 [26]. Total carbon (TC) content was determined using high-temperature decomposition with infrared detection of TOC in a multi-NC 3100 analyzer (Analytik Jena, Jena, Germany). Contents of total nitrogen (TN), ammonia nitrogen, orthophosphates, and COD in the sludge supernatant were determined with the spectrophotometric method after previous mineralization using a Hach DR6000 spectrometer (Hach, Loveland, CO, USA). The supernatant was obtained by AGS centrifugation in an MPW-251 laboratory centrifuge (MPW Med. Instruments, Warsaw, Poland) at a rotational speed of 5000 rpm for 10 min. The potentiometric method was used to determine pH. Biogas composition was controlled at the end of the process using a DP-28BIO gas analyzer (Nanosens, Wysogotowo, Poland).

### 2.5. Molecular Methods

The FISH method was deployed to identify the consortia of anaerobic microorganisms. Four molecular probes were used for hybridization, namely a universal probe for EUB338 bacteria and ARC915 archaea and a specific probe for *Methanosarcinaceae* MSMX860 and *Methanosaeta* MX825. The samples were analyzed under an epifluorescence microscope with a 100× objective and 1000× total magnification (Nikon, Tokyo, Japana). The population numbers of microorganisms of the tested species were calculated from cells stained with DAPI using Image Processing and Analysis in Java (ImageJ) software developed by the National Institutes of Health and Laboratory for Optical and Computational Instrumentation (LOCI, University of Wisconsin, Madison, WI, USA) [27]. 

### 2.6. Computation Methods

The biogas production rate (r) and reaction rate constants (k) were determined in all experimental variants based on test data obtained with the non-linear regression method using Statistica 13.3 PL software (Statsoft, Inc., Tulsa, OK, USA). A non-linear regression iterative method was used, in which the function is replaced by a linear differential with respect to the determined parameters in each iterative step. The contingency coefficient φ2 was adopted as a measure of the curve’s fit to the test data. It was assumed that the model was fitted to the experimental points where the value of this coefficient did not exceed 0.2 (Statistica 13.3 PL package (Statsoft, Inc., Tulsa, OK, USA)).

The specific energy input (Es) was calculated using Equation (1) (data for computing the power of the SCO_2_ generator (P), SCO_2_ mass (M), and SCO_2_ generator yield (Y) were adopted for a P3000 Pelletizer device (Cold Jet, Loveland, CO, USA) [28]):

(1) $$E_{s}=P_{{\mathrm{SCO}}_{2}\;}\cdot M_{{\mathrm{SCO}}_{2}}/Y_{{\mathrm{SCO}}_{2}}\;(\mathrm{kWh})\;$$

where:

P_SCO2_—power of SCO_2_ generator (W);
M_SCO2_—mass of SCO_2_ (kg);
Y_SCO2_—yield of SCO_2_ generator (kg/h).

The energy output (*E_out_*) generated from methane production was calculated using Equation (2):

(2) $$E_{\mathrm{out}}=Y_{\mathrm{Methane}}\cdot {\mathrm{CV}}_{\mathrm{Methane}}\;\cdot M_{\mathrm{AGS}}(\mathrm{Wh})\;$$

where:

Y_Methane_—methane yield (dm^3^/kg fresh matter (FM));
CV_Methane_—calorific value of methane (Wh/dm^3^);
M_AGS_—mass of AGS (kg).

The net energy output (E_nout_) was calculated using Equation (3):

(3) $$E_{\mathrm{nout}}=E_{\mathrm{out}\left(\mathrm{Vx}\right)}-E_{\mathrm{out}\left(v1\right)}\;(\mathrm{Wh})\;$$

where:

E_out(Vx)_—the energy output in n-variant (Wh);
E_out(V1)_—the energy output in V1 (Wh).

The net energy gain (E_net_) was calculated from Equation (4):

(4) $$E_{\mathrm{net}}=E_{\mathrm{nout}}-E_{S}\;(\mathrm{Wh})\;$$

where:

E_nout_—the net energy output (Wh);
E_s_—the specific energy input (Wh).

The energy value (EV) was computed using Equation (5):

(5) $$\mathrm{EV}=E_{\mathrm{net}}\cdot \mathrm{EP}\;\left[€\;\right]$$

where:

Enet—the net energy gain (Wh);
EP—energy price (EUR/Wh)—the energy price was adopted as the mean from 2020 to the first half of 2022 based on Eurostat data [29].

The SCO_2_ value (SCO_2_V) was computed from Equation (6):

(6) $${\mathrm{SCO}}_{2}V=M_{{\mathrm{SCO}}_{2}}\cdot \mathrm{CPP}\;\left[€\;\right]$$

where:

M_SCO2_—mass of SCO_2_ (kg);
CPP—price of EU Carbon Permits (EUR/kg)—the price of EU Carbon Permits was adopted as the mean from 2020 to the first half of 2022 based on Trading Economics data [30].

The profit was computed according to Equation (7):

(7) $$\mathrm{Profit}=\mathrm{EV}+{\mathrm{SCO}}_{2}V\;\left[€\;\right]$$

where:

EV—energy value (EUR);
SCO_2_V—SCO_2_ value (EUR).

### 2.7. Statistical and Optimization Methods

All experimental variants were conducted in triplicate. The statistical analysis of the results was carried out with the Statistica 13.3 PL package (Statsoft, Inc., Tulsa, OK, USA). The Shapiro–Wilk test was used to verify the hypothesis regarding the distribution of every researched variable. The ANOVA test was performed to establish the significance of differences between variables. Levene’s test was used to check the homogeneity of variance in groups, and Tukey’s HSD test was used to determine the significance of differences between the analyzed variables. Differences were found significant at α = 0.05.

Empirical equations were elaborated using stepwise regression with multiple regression. Key predictors of changes in the values of the estimated parameters were identified in model systems. Model fit to empirical data was verified by means of the determination coefficient (Statistica 13.3 PL package (Statsoft, Inc., Tulsa, OK, USA)).

## 3. Results and Discussion

### 3.1. Stage 1

**Figure 5:**
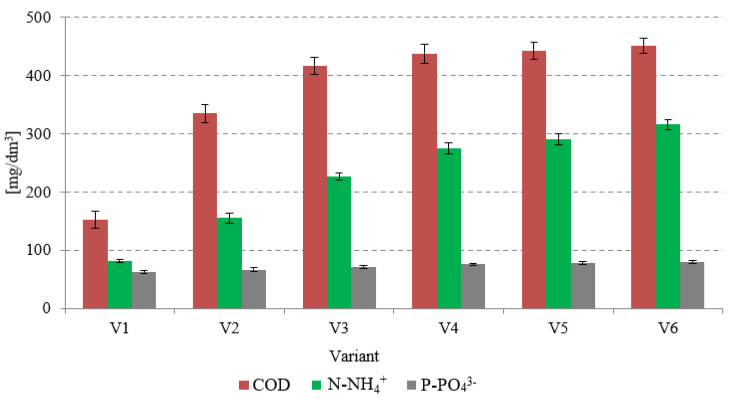
Changes in concentrations of organic compounds and nutrients in AGS supernatant caused by the pre-treatment with SCO_2_.

Investigations conducted so far have proved that damage to CAS cell structures triggered by the treatment with SCO_2_ may increase the concentration of dissolved COD, proteins, molecular material, orthophosphates, and ammonia nitrogen in the supernatant [25]. These phenomena contribute to the increased turbidity of the supernatant and improve CAS susceptibility to dehydration [31]. Effective CAS disintegration has also been proved by means of FTIR spectroscopy [21]. The increase in COD concentration is claimed to be related to the breaking of CAS structures followed by the degradation of single cells of microorganisms [32]. Analogous effects were observed in the present study during AGS pre-treatment using SCO_2_. In the case of the non-pre-treated sludge (V1), the COD concentration in the supernatant was 152 ± 14 mgO_2_/dm^3^ (Figure 5). In variants V2–V4, the COD concentration was observed to increase successively from 334 ± 15 mgO_2_/dm^3^ (V2) to 437 ± 16 mgO_2_/dm^3^ (V4). A further increase in the SCO_2_ dose caused no statistically significant (*p* > 0.5) changes in the COD concentrations in the supernatant (Figure 5), which reached 442 ± 15 mgO_2_/dm^3^ (V5) and 450 ± 13 mgO_2_/dm^3^ (V6). 

Machnicka et al. (2019) [21] observed a correlation between the SCO_2_/CAS ratio and COD concentration in the supernatant. Pre-treatment caused the COD concentration to increase from 63 mgO_2_/dm^3^ in the raw sludge to 205 mgO_2_/dm^3^ in the sludge pre-treated at the SCO_2_/CAS ratio of 1:0.25. Increasing SCO_2_/CAS to 1:1 caused an increase in COD concentration to 889 mgO_2_/dm^3^ [21]. Additionally, Zawieja (2018) [33] noted a significant COD increase in the supernatant. At SCO_2_/CAS ratios ranging from 0.05/1.0 to 0.75/1.0, they observed changes in the COD concentration ranging from 119 mgO_2_/dm^3^ to 296 mgO_2_/dm^3^ [33]. Another research [25] demonstrated a proportional increase in the COD concentration in a dairy CAS supernatant along with an increase in the SCO_2_/CAS volume ratio to 0.3. In crude CAS, the COD concentration was at 400.5 ± 23.8 mg/dm^3^. The highest COD values, falling within a narrow range of 490.6 ± 12.9 to 510.5 ± 28.5 mg/dm^3^, were noted at SCO_2_/CAS ratios ranging from 0.3 to 0.5 [25]. Stabnikova et al. (2008) [34] demonstrated an almost two-fold increase in COD concentration in the supernatant in their study investigating the effect of the freezing/thawing process on food waste. In turn, Bailey et al. (2011) [35] reported a 15% increase in COD concentration when freezing/thawing excess municipal sewage sludge. 

Damage caused by SCO_2_ to the cellular structure of microorganisms triggers the release of enzymes contained in their protoplasts, whose hydrolytic activity results in the degradation of organic compounds of nitrogen and phosphorus and consequently in increased concentrations of ammonia nitrogen and orthophosphates in the supernatant [36]. In the present study, an increase in the SCO_2_ dose was accompanied by increasing concentrations of N-NH_4_^+^ and P-PO_4_^3−^. In V2, the N-NH_4_^+^ concentration reached 155 ± 8.4 mg/dm^3^ and that of P-PO_4_^3−^ reached 66.5 ± 3.5 mg/dm^3^. In the supernatant of raw AGS, the respective values were 81.5 ± 3.1 mg N-NH_4_^+^/dm^3^ and 62.2 ± 2.2 mg P-PO_4_^3−^/dm^3^ (Figure 5). The applied SCO_2_ dose had a significant effect on the N-NH_4_^+^ and P-PO_4_^3−^ concentrations determined in variants V1–V4, which were observed to ultimately increase to 274 ± 9.3 mg/dm^3^ and 75.7 ± 1.9 mg/dm^3^, respectively (Figure 5). In the subsequent variants, the increase in their concentrations was no longer statistically significant (*p* > 0.5) (Figure 5).

In the study conducted by Zawieja (2019) [37], the N-NH_4_^+^ concentration in the supernatant of crude CAS approximated 43 mg/dm^3^ and increased successively along with an increasing SCO_2_ dose, reaching ca. 102 mg/dm^3^ at the SCO_2_/CAS volume ratio of 0.75/1.0 [37]. In another study [25], the pre-treatment of dairy CAS with SCO_2_ increased N-NH_4_^+^ concentration in the supernatant from 155.2 ± 10.2 mg/dm^3^ in the crude sludge to 185.9 ± 11.1 mg/dm^3^ in the sludge pre-treated at the SCO_2_/CAS volume ratio of 0.5. Likewise, an increasing SCO_2_ dose caused the P-PO_4_^3−^ concentration to increase from 198.5 ± 23.1 to 300.6 ± 35.9 mg/dm^3^ [25]. In their experiment with freezing/thawing mixed sewage sludge, Montusiewicz et al. (2010) [38] reported an increase in the N-NH_4_^+^ concentration in the supernatant from 94.0 mg/dm^3^ in the non-conditioned sludge to 130.9 mg/dm^3^ as well as an over two-fold increase in P-PO_4_^3−^ concentration from 86.4 mg/dm^3^ in the control sample to 185.2 mg/dm^3^. In another study, this method of conditioning municipal CAS resulted in a 1.5-fold to 2.5-fold increase in the P-PO_4_^3−^ concentration in the supernatant [39]. An expected technological outcome of pre-treatment is the increased effectiveness of methane fermentation [40]. Undoubtedly, the disintegration of complex macromolecules of the biomass, followed by the efficient transfer of organic compounds to the dissolved phase, increases substrate availability to anaerobic bacteria [41]. 

### 3.2. Stage 2

#### 3.2.1. Biogas and Methane Production

**Figure 6:**
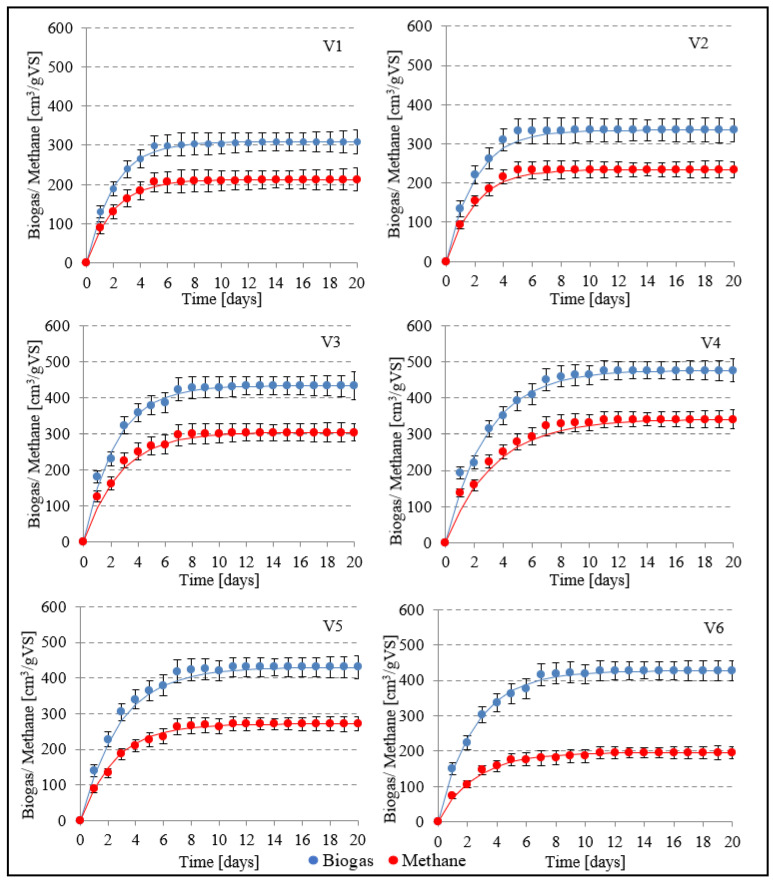
The course of biogas and methane production processes in experimental variants.

**Figure 7:**
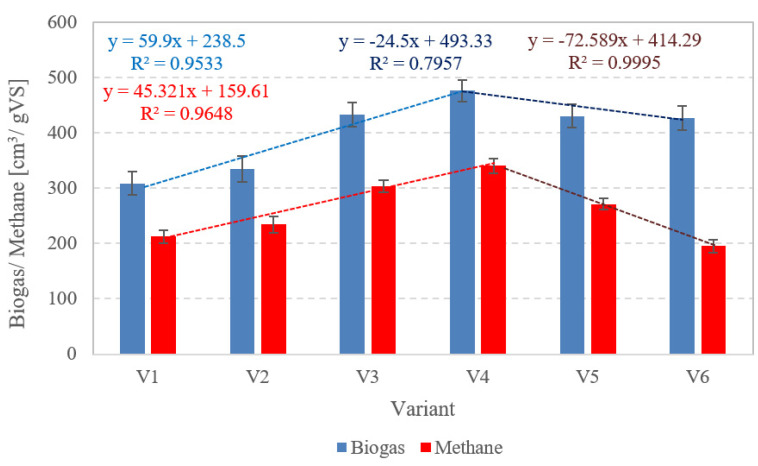
Biogas and methane production in particular technological variants.

The biogas yield of raw AGS (V1) was 309 ± 21 cm^3^/gVS (Figure 6 and Figure 7). The r value was 47.7 cm^3^/d, and the CH_4_ concentration in the biogas produced was 68.84 ± 2.2% (Table 2). The highest yields of biogas and methane were obtained in V4, i.e., 476 ± 20 cm^3^/gVS and 341 ± 13 cm^3^/gVS, respectively. The rate of the process reached 113.3 cm^3^/d biogas, and CH_4_ concentration was at 71.58 ± 1.7% (Figure 6 and Figure 7). Compared to V1, an increase in biogas and CH_4_ production yield in this variant reached 54.05 ± 3.5% and 60.18 ± 2.4%, respectively. The subsequent variants brought about an AD yield reduction and a significant decrease in CH_4_ concentration, whose values reached 430 ± 21 cm^3^/gVS biogas and 271 ± 10 cm^3^/gVS CH_4_ (63.03 ± 1.3%) in V5, as well as 427 ± 22 cm^3^/gVS biogas and 196 ± 12 cm^3^/gVS methane in V6 (45.80 ± 2.1%) (Figure 6 and Figure 7).

A study conducted by Bernat et al. (2017) [42] demonstrated that the potential of biogas production from AGS was 1.8-fold lower than from CAS. At the applied OLRs ranging from 2.0 to 6.0 gVS/cm^3^·d, these authors observed that biogas productivity decreased along with increasing OLR, i.e., from 408.9 cm^3^/gVS at OLR 2 gVS/cm^3^·d to 318.5 cm^3^/gVS at OLR 6 gVS/cm^3^·d, and that the CH_4_ concentration in the biogas ranged from 56.7 to 59.5% [42]. In turn, Cydzik-Kwiatkowska et al. (2022) [43] applied the ultrasound pre-treatment of AGS to boost biogas production. In the case of AGS not subjected to ultrasonic disintegration, they found no significant differences in the biogas yield and the CH_4_ content of biogas in the analyzed OLR range from 1.0 to 3.0 gVS/cm^3^·d. The biogas yield approximated 375 cm^3^/gVS, and the CH_4_ concentration in the biogas ranged from 56.7 ± 0.4 to 57.5 ± 0.6%. Biogas production was observed to increase significantly along with the extension of the disintegration process. Regardless of OLR, after 0.5, 4.0, and 8.0 min of disintegration, the biogas yield was ca. 400 cm^3^/gVS, 420 cm^3^/gVS, and 455 cm^3^/gVS, respectively [43]. Zawieja (2019) [37] investigated the effect of pre-treatment using SCO_2_ on the course of methane fermentation of modified sewage sludge. At the SCO_2_ to excess sludge volume ratio of 0.55/1, this author achieved a biogas yield of 620 cm^3^/gVS. In the case of prepared sludge, the CH_4_ content of the biogas approximated 78% [37]. In turn, Nowicka et al. (2014) [44] used SCO_2_ for the disintegration of municipal CAS prior to AD. In the most effective variant, biogas yield was 49% higher compared to raw CAS [44].

#### 3.2.2. pH and FOS/TAC

The use of SCO_2_ caused a decrease in the pH value of AGS (Table 3). In V1, the pH reached 7.80 ± 0.1 and successively decreased to 6.93 ± 0.1 in V4. A further increase in the SCO_2_ dose in the subsequent experimental variants reduced the pH value of AGS to 6.42 ± 0.1 in V5 and 6.31 ± 0.1 in V6. During CO_2_ sublimation, its part dissolves in the supernatant. CO_2_ is well soluble in aqueous solutions, and its solubility at a temperature of 25 °C reaches 2900 mg/dm^3^ [45]. Its sublimation results in the formation of carbonate ions (CO_3_^2−^), bicarbonate ions (HCO_3_^−^), and hydrogen ions (H^+^), leading to pH reduction [46], which in turn negatively affects the outcomes of methane fermentation expressed by biogas and methane yields [38]. Hence, the pH values measured in digesters were a consequence of initial conditions after pre-treatment with SCO_2_. In the control sample in V1, the pH value measured in digesters decreased from 7.48 ± 0.1 to 7.01 ± 0.1 after AD (Table 3). In V2–V4, the pH ranged from 7.12 ± 0.1 to 7.36 ± 0.1 and decreased after AD to values ranging from 6.75 ± 0.1 to 6.86 ± 0.1 (Table 3). In V5 and V6, in which the pre-treatment contributed to environment acidification, pH values measured in digesters decreased drastically after AD, i.e., from 6.93 ± 0.1 to 6.44 ± 0.1 in V5 and from 6.89 ± 0.1 to 6.30 ± 0.1 in V6 (Table 3). This pH decrease was reflected in the population of methanogens and, consequently, in CH_4_ yield. In another study [37], the pH of digesters was also observed to decrease upon sludge pre-treatment with SCO_2_, which resulted in diminished AD yield. It is all the more important that the pH value in an anaerobic digester is a key factor determining process stability [47]. 

The FOS/TAC ratio was sustained at the optimal level in all experimental variants. Its lowest value was determined in V6, i.e., 0.40 ± 0.02, and its highest value was sustained in V1, i.e., 0.42 ± 0.04 (Table 3). These differences were not statistically significant (*p* > 0.5). The FOS/TAC ratio is a value describing the ratio of the volatile organic acid to the alkaline buffer capacity. It is often applied to assess process stability in anaerobic digesters. According to literature data [48,49], the FOS/TAC values of a stable AD process range from 0.2 to 0.6. The FOS/TAC values exceeding 0.6 are indicative of operating conditions inappropriate for anaerobic microorganisms and contribute to biogas yield decline [48].

#### 3.2.3. Bacterial Community Structure

This part of the study analyzed the effect of experimental variants on the taxonomic structure of the population of anaerobic bacteria (Table 4). In V1, the prevailing taxonomic group was Bacteria (EUB338), accounting for 69 ± 10% of the anaerobes population (Table 4). In turn, methanogenic archaea (ARC915) accounted for 24 ± 6%, *Methanosarcinaceae* (MSMX860) accounted for 10 ± 4%, and *Methanosaeta* (MX825) accounted for 5 ± 2% (Table 4). In the subsequent variants, again, Bacteria (EUB338) turned out to be the prevailing consortium of microorganisms, with their percentage in the population of anaerobes ranging from 68 ± 10% in V6 to 70 ± 11% in V2, regardless of SCO_2_ dose (Table 4). In V2–V4, the contribution of Archaea (ARC915) in the population of anaerobes ranged from 24 ± 4 to 25 ± 11%, that of *Methanosarcinaceae* (MSMX860) ranged from 11 ± 4 to 12 ± 5%, and that of *Methanosaeta* (MX825) ranged from 8 ± 3 to 9 ± 4% (Table 4). In V5 and V6, the contribution of Archaea (ARC915) diminished to 19 ± 9% and 18 ± 8%, respectively. The contribution of *Methanosarcinaceae* (MSMX860) reached 11 ± 4% in V5 and 10 ± 6% in V6, whereas that of *Methanosaeta* (MX825) accounted for 7 ± 3% in V5 and for 5 ± 2% in V6 (Table 4). This decrease observed in V5–V6 in the abundance of the selected consortia was due to environment acidification prior to digestion, which influenced environmental conditions as well as the course and effectiveness of the AD process in digesters. After AD, the pH value reached barely 6.44 ± 0.1 in V5 and 6.30 ± 0.1 in V6. Hence, it may be concluded that the changes in the population of methanogens were ascribed to the modification of environmental conditions upon pre-treatment with SCO_2_. Fermentative bacteria may effectively function in a broad range of pH values, i.e., between 4.0 and 8.0, whereas methanogenic bacteria are functionally active in the pH range of 6.5–7.5 [50].

#### 3.2.4. Empirical Models and Correlations

One of the ways to assess the effectiveness of pre-treatment processes is to analyze concentrations of selected indicators in the dissolved phase of the organic substrate undergoing disintegration [51]. Usually, this assessment is made based on monitoring concentrations of organic compounds [52]. In some cases, it is possible to develop reliable correlations and models to estimate AD efficiency based on the presence of organic compounds in the dissolved phase [53]. For this reason, carrying out this type of assessment is useful from a practical point of view, as it reduces the need to conduct more advanced measurements in order to determine the effectiveness of the applied pre-treatment methods. 

**Figure 8:**
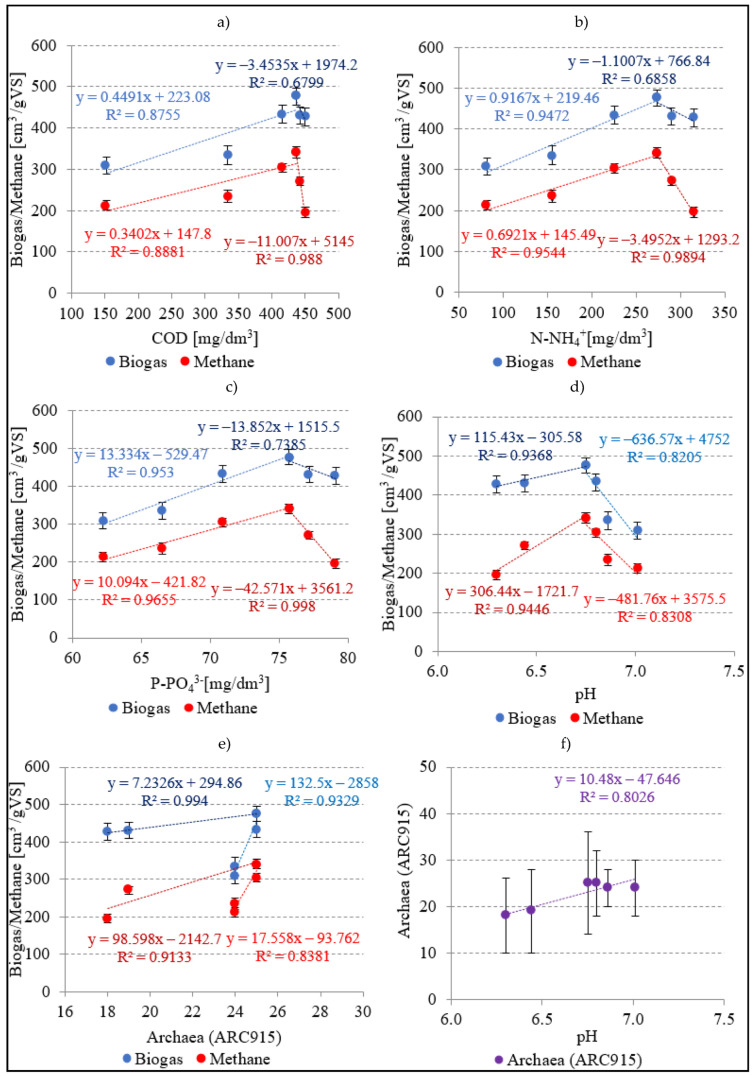
Correlations between: (**a**) COD, (**b**) N-NH_4_^+^, and (**c**) P-PO_4_^3−^ concentrations in the dissolved phase, (**d**) pH, (**e**) Archaea, and the yields of biogas and methane, as well as between (**f**) pH and Archaea.

In variants from V1 to V4, very strong positive correlations were found between the concentrations of COD (Figure 8a), N-NH_4_^+^ (Figure 8b), and P-PO_4_^3−^ (Figure 8c) in the dissolved phase and biogas production yield. The coefficients of determination reached R^2^ = 0.8755, R^2^ = 0.9472, and R^2^ = 0.953, respectively. The higher SCO_2_ doses tested in V5 and V6 resulted in negative correlations between concentrations of the monitored indicators in the dissolved phase and biogas yield, i.e., R^2^ = 0.6799 for COD, R^2^ = 0.6858 for N-NH_4_^+^, and R^2^ = 0.7385 for P-PO_4_^3−^ (Figure 8). Similar phenomena were observed for the concentrations of COD (Figure 8a), N-NH_4_^+^ (Figure 8b), and P-PO_4_^3−^ (Figure 8c) in the dissolved phase and for the CH_4_ yield. Variants V1–V4 were characterized by very strong positive correlations between the concentrations of COD (R^2^ = 0.8881), N-NH_4_^+^ (R^2^ = 0.9544), and P-PO_4_^3−^ (R^2^ = 0.9655) in the dissolved phase and CH_4_ yield (Figure 8). Analyses conducted in the subsequent variants demonstrated very strong negative correlations expressed by R^2^ = 0.988, R^2^ = 0.9894, and R^2^ = 0.998, respectively (Figure 8).

In V1–V4, strong negative correlations were noted between pH values after AD and yields of biogas (R^2^ = 0.8205) (Figure 8d) and CH_4_ (R^2^ = 0.8305) (Figure 8d), as well as a strong correlation between the Archaea percentage in the population of anaerobic bacteria and CH_4_ yield (R^2^ = 0.8381) (Figure 8e). A strong correlation was also found between pH value and Archaea percentage, with a determination coefficient reaching R^2^ = 0.8026 (Figure 8f). In variants V1–V4, the taxonomic structure of the population of fermentative bacteria was similar, whereas an increase in biogas and CH_4_ yields was due to increased availability and improved biodegradability of the substrate after pre-treatment. In variants V5–V6, availability and biodegradability were similar to those noted in V4, as indicated by concentrations of the indicators tested in the dissolved phase, whereas high applied SCO_2_ doses decreased the pH value, thereby inhibiting the populations of methanogenic bacteria. These changes were indicated by strong correlations noted between Archaea and CH_4_ yield as well as between pH value and Archaea. Methanogenic bacteria are sensitive to changes in the environment and require neutral pH for their optimal metabolic activity [54].

**Figure 9:**
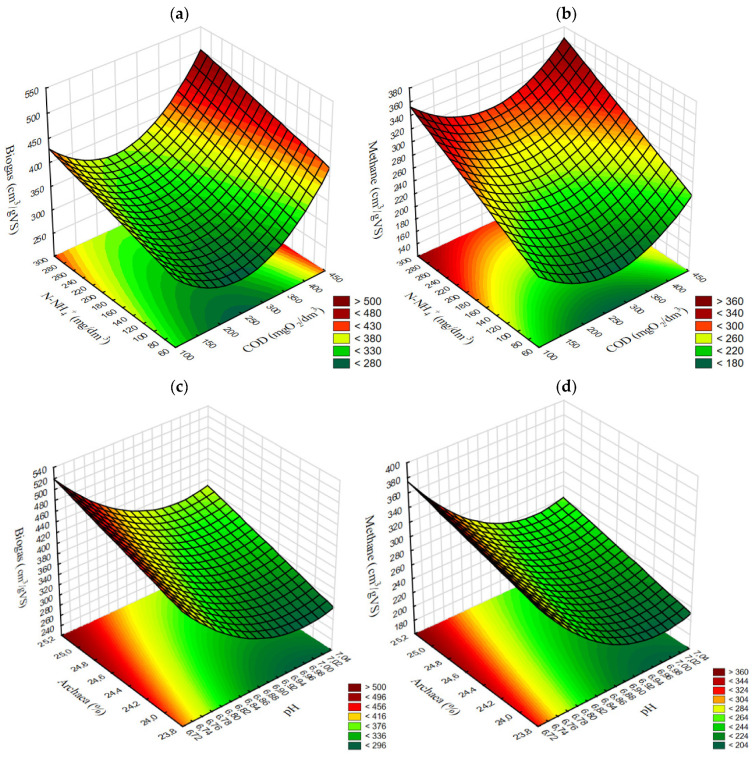
Surface correlation between COD and N-NH_4_ concentrations and the yields of (**a**) biogas and (**b**) methane; and between pH and Archaea contribution in the population of methanogens and the yields of (**c**) biogas and (**d**) methane.

The results achieved in variants V1–V4 allowed presenting a correlated surface effect of COD and N-NH_4_^+^ concentrations in the supernatant (Figure 9a,b) as well as the effect of pH value and Archaea percentage in the populations of methanogens (Figure 9c,d) on the yields of biogas and methane. 

The multiple regression method was deployed to develop empirical equations for biogas and methane yield estimation. Variants V1–V4 were considered in the estimation because of the revealed linear correlations. It was found that biogas and methane yields were statistically significantly (*p* < 0.5) affected by such dependent variables as COD and N-NH_4_^+^ concentrations in the dissolved phase as well as the SCO_2_/AGS volume ratio. The postulated model of biogas yield (8) is characterized by an estimation error of ±13.446 and reflects ca. 96.69% of changes in the process of biogas production (R^2^ = 0.9669). The methane yield model (9) reflects ca. 98.04% changes in the process of its production (R^2^ = 0.9804) with an estimation error of ±7.6996.

(8) $$\mathrm{BIOGAS}=-0.69\mathrm{COD}+4.03N-{\mathrm{NH}}_{4}^{+}-1359.45{\mathrm{SCO}}_{2}/\mathrm{AGS}+78.8$$

(9) $$\mathrm{METHANE}=-0.458\mathrm{COD}+2.525N-{\mathrm{NH}}_{4}^{+}-747.830{\mathrm{SCO}}_{2}/\mathrm{AGS}+73.176$$

where:

BIOGAS—biogas yield, cm^3^/gVS;
METHANE—methane yield, cm^3^/gVS;
COD—COD concentration in the supernatant, mgO_2_/dm^3^;
N-NH_4_^+^—N-NH_4_^+^ concentration in the supernatant, mg/dm^3^;
SCO_2_/AGS—volume ratio of SCO_2_ to AGS.

#### 3.2.5. Energy and Economic Balance

**Figure 10:**
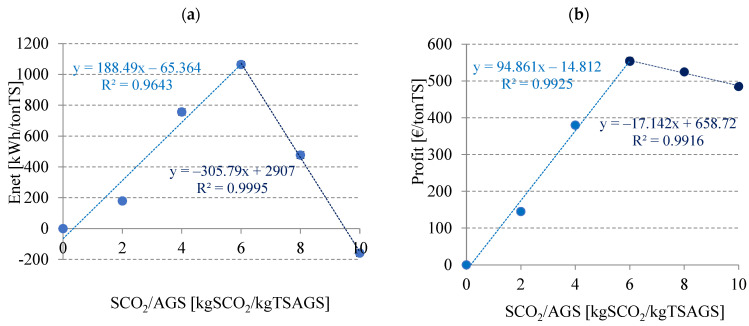
Correlations between the SCO_2_/AGS mass ratio and (**a**) Enet, and (**b**) Profit.

The assessment of energetic effectiveness is extremely important to the evaluation of process viability, especially on a large scale [55]. Considering the production yield of methane and its energy value per volume reaching 9.17 Wh/dm^3^, the highest gross energy gain was achieved in V4, i.e., 218.49 ± 1.6 Wh (Table 5). In the control variant (V1), the gain was only 136.48 ± 1.5 Wh. Energy consumption for the pre-treatment was found to be proportional to SCO_2_ dose and ranged from 0.64404 Wh in V2 to 3.22018 Wh in V6 (Table 5). Positive net energy gains were obtained in variants V2–V4. The gain increased from 13.45 ± 1.4 Wh in V2 to 80.08 ± 1.5 Wh in V4. In the subsequent variants, the net energy gains were observed to decrease drastically, reaching 34.59 ± 1.7 Wh in V5 and −14.11 ± 1.6 Wh in V6 (Table 5). When converted per tons of TS, the optimal variant produced net energy gain at 1047.85 ± 20 Wh/MgTS (Table 5). In V1–V4, very strong positive correlations were found between the SCO_2_/AGS mass ratio and net energy gain (R^2^ = 0.9643) (Figure 10a). The use of this type of pre-treatment to intensify methane fermentation could be even more advisable if a closed CO_2_ circuit was applied in the following cycle: biogas production—biogas enrichment—SCO_2_ production—sludge disintegration—digestion—biogas production. It is an important argument that affects the improvement in the economic and technological viability of fermentation processes and provides a solution for reducing CO_2_ emissions to the atmosphere, being necessary from the environmental protection standpoint. The estimated economic analysis demonstrated a feasible profit ranging from 144.99 EUR/tonTS in V2 to 554.05 EUR/tonTS in V4 (Table 6). Very strong positive correlations were found in V1–V4 between the SCO_2_/AGS mass ratio and profit (R^2^ = 0.9925) (Figure 10b). Energy sustainability is one of the critical parameters that need to be analyzed to ensure the successful application of pre-treatment processes [56]. Balasundaram et al. (2022) [57] made a critical assessment of the energetic effectiveness of various energy-consuming techniques of sewage sludge pre-treatment. They demonstrated that the conventional heat pre-treatment of sludge (∼5% TS) ensured 244 cm^3^CH_4_/gTS, which could yield a positive energy balance at 2.6 kJ/kg TS, and that the microwave pre-treatment allowed generating only 178 cm^3^CH_4_/gTS, yielding a negative energy balance at −15.62 kJ/kg TS. In turn, the ultrasound pre-treatment of sewage sludge prior to AD required sludge thickening to achieve a positive energy balance [58]. Therefore, the proposed pre-treatment method with the use of SCO_2_ seems very promising.

## 4. Conclusions

The present study demonstrated a proportional increase in COD, N-NH_4_^+^, and P-PO_4_^3−^ concentrations in the supernatant, along with increasing doses of SCO_2_ within the applied SCO_2_/AGS volume ratios ranging from 0.1 to 0.3. The higher SCO_2_ dose tested had no significant effect upon increasing concentrations of the analyzed indicators in the dissolved phase. 

The highest unit biogas yield, I.e., 476 ± 20 cm^3^/gVS, was achieved in the variant with an SCO_2_/AGS volume ratio of 0.3. Methane yield in this variant reached 341 ± 13 cm^3^/gVS. Increasing the SCO_2_ dose caused no significant changes in the volumes of biogas and methane produced. Optimization procedures demonstrated COD and N-NH_4_^+^ concentrations, as well as the SCO_2_/AGS ratio, to be the significant predictors of changes in the values of the estimated parameters, i.e., biogas and methane yields.

The study proved that applying SCO_2_ doses higher than 0.3 contributed to a significant decrease in the pH value of AGS and environment acidification, thereby directly affecting a reduction in the percentage of methanogenic bacteria in the anaerobic bacterial community, which ultimately decreased the CH_4_ content of the biogas produced.

The energetic analysis demonstrated the highest net energy gain, reaching 1047.85 ± 20 kWh/tonTS, in the variant with the SCO_2_/AGS volume ratio of 0.3. In turn, the economic analysis proved the feasibility of achieving a profit of 554.05 EUR/tonTS in this variant. The use of SCO_2_ from the produced biogas could significantly improve the energy balance. Its production via technologies dedicated to biogas upgrading and its application for sewage sludge pre-treatment correspond with the idea of material recycling and directly inscribe into the assumptions of the circular economy. This approach also supports the idea of reducing carbon dioxide emissions through its sequestration and use in a closed cycle.

## Figures and Tables

**Table 1:** Characteristics of AGS and AS inoculum used in the experiment.

Indicator	Unit	CAS	AGS	AS
pH	-	7.61 ± 0.1	7.80 ± 0.1	7.27 ± 0.2
Total solids (TS)	(%)	4.41 ± 0.1	7.42 ± 0.1	3.37 ± 0.1
Volatile solids (VS)	(%TS)	82.62 ± 3.5	90.17 ± 5.8	66.43 ± 6.1
Mineral solids (MS)	(%TS)	17.38 ± 1.3	9.83 ± 1.1	33.57 ± 5.4
Total carbon (TC)	(mg/gTS)	608 ± 19	652 ± 21	313 ± 12
Total organic carbon (TOC)	(mg/gTS)	578 ± 17	606 ± 16	300 ± 10
Total nitrogen (TN)	(mg/gTS)	95 ± 6.2	100 ± 5.4	33.4 ± 3.6
C/N ratio	-	6.4 ± 0.1	6.5 ± 0.1	9.37 ± 0.3
Total phosphorus (TP)	(mg/gTS)	3.2 ± 1.1	6.9 ± 1.4	1.8 ± 0.1

**Table 2:** Rates of biogas and methane production processes (r), reaction rate constants (k), and methane concentration.

Variant 1	Biogas		Methane		
	r	k	%CH_4_	r	k
	(cm^3/^d)	(1/d)	(%)	(cm^3/^d)	(1/d)
1	47.7	0.15	68.84 ± 2.2	22.6	0.11
2	56.1	0.17	70.00 ± 2.1	27.5	0.12
3	93.7	0.22	70.14 ± 1.8	46.1	0.15
4	113.3	0.24	71.58 ± 1.7	58.1	0.17
5	92.5	0.22	63.03 ± 1.3	36.7	0.14
6	91.2	0.21	45.80 ± 2.1	19.1	0.10

**Table 3:** Changes in pH values upon pre-treatment and AD and FOS/TAC after AD.

	V1	V2	V3	V4	V5	V6
pH of AGS after pre-treatment with SCO_2_	7.80 ± 0.1	7.51 ± 0.1	7.21 ± 0.1	6.93 ± 0.1	6.42 ± 0.1	6.31 ± 0.1
pH of AGS + inoculum	7.48 ± 0.1	7.36 ± 0.1	7.24 ± 0.1	7.12 ± 0.1	6.93 ± 0.1	6.89 ± 0.1
pH after AD	7.01 ± 0.1	6.86 ± 0.1	6.80 ± 0.1	6.75 ± 0.1	6.44 ± 0.1	6.30 ± 0.1
FOS/TAC	0.42 ± 0.04	0.42 ± 0.03	0.41 ± 0.04	0.41 ± 0.05	0.40 ± 0.03	0.40 ± 0.02

**Table 4:** Microbial taxonomy in particular experimental variants.

Taxonomic Group	V1	V2	V3	V4	V5	V6
Bacteria (EUB338)	69 ± 10	70 ± 11	69 ± 12	69 ± 10	69 ± 11	68 ± 10
Archaea (ARC915)	24 ± 6	24 ± 4	25 ± 7	25 ± 11	19 ± 9	18 ± 8
*Methanosarcinaceae* (MSMX860)	10 ± 4	11 ± 4	12 ± 4	12 ± 5	11 ± 4	10 ± 6
*Methanosaeta* (MX825)	5 ± 2	8 ± 3	8 ± 3	9 ± 4	7 ± 3	5 ± 2

**Table 5:** Energy balance.

Variant	SCO_2_/AGS	ρ_AGS_	M_AGS_	V_AGS_	ρ_SCO2_	V_SCO2_	M_SCO2_	P_SCO2_	W_SCO2_	E_s_	Y_methane_	Y_methane_	CV_methane_	E_out_	E_nout_	E_net_	E_net_
	-	kg/dm^3^	kg	dm^3^	kg/dm^3^	dm^3^	kg	M	kg/h	Wh	dm^3^/kgVS	dm^3^/kgFM	Wh/dm^3^	Wh	Wh	Wh	kWh/tonTS
1	0	1.03	1.03	1	1.56	0	0	4500	1090	0	213 ± 12	14.88 ± 1.5	9.17	136.48 ± 1.5	0	0	0
2	0.1					0.1	0.156			0.64404	235 ± 15	16.42 ± 2.2		150.58 ± 2.2	14.10 ± 1.4	13.45 ± 1.4	176.02 ± 18
3	0.2					0.2	0.312			1.28807	304 ± 11	21.24 ± 1.8		194.79 ± 1.8	58.31 ± 1.6	57.02 ± 1.6	746.08 ± 21
4	0.3					0.3	0.468			1.93211	341 ± 13	23.83 ± 1.6		218.49 ± 1.6	82.02 ± 1.5	80.08 ± 1.5	1047.85 ± 20
5	0.4					0.4	0.624			2.57615	271 ± 10	18.94 ± 2.0		173.64 ± 2.0	37.16 ± 1.7	34.59 ± 1.7	452.56 ± 22
6	0.5					0.5	0.78			3.22018	196 ± 12	13.70 ± 1.8		125.59 ± 1.8	−10.89 ± 1.6	−14.11 ± 1.6	−184.66 ± 21

SCO_2_/AGS—volume ratio of SCO_2_ to AGS; ρ_AGS_—specific density of AGS; M_AGS_—mass of AGS; V_AGS_—volume of AGS; ρ _SCO2_—density of SCO_2_; V _SCO2_—volume of SCO_2_; M_SCO2_—mass of SCO_2_; P_SCO2_—generator power SCO_2_; W_SCO2_—efficiency of SCO_2_ generator; E_s_—specific energy input; Y_methane_—methane yield; CV_methane_—methane calorific value; E_out_—energy output; E_nout_—net energy output; E_net_—net energy gain.

**Table 6:** Estimated economic analysis.

Variant	E_net_	Energy Price	Energy Value	Price of EU Carbon Permits	SCO_2_	Value of SCO_2_	Profit	Profit
	Wh	EUR/kWh	EUR	EUR/kg	kg	EUR	EUR	EUR/tonTS
1	0	0.2266	0.00000	0.0517	0	0	0	0
2	13.45 ± 1.4		0.00302		0.156	0.00807	0.01108	144.99
3	57.02 ± 1.6		0.01291		0.312	0.01613	0.02905	380.04
4	80.08 ± 1.5		0.01815		0.468	0.02420	0.04234	554.05
5	34.59 ± 1.7		0.00788		0.624	0.03226	0.04014	525.24
6	−14.11 ± 1.6		−0.00322		0.78	0.04033	0.03710	485.48

## Data Availability

Not applicable.
